# Sustainable Augmentation of Chickpea Protein Functionality and In Vitro Digestion via Spent Coffee Phenolics: A Protein–Phenolic Interaction Study

**DOI:** 10.1002/fsn3.70914

**Published:** 2025-11-21

**Authors:** Beyza Saricaoglu, Busra Gultekin Subasi, Esra Capanoglu

**Affiliations:** ^1^ Department of Food Engineering, Faculty of Chemical and Metallurgical Engineering Istanbul Technical University Istanbul Türkiye; ^2^ Research Group for Food Production Engineering, National Food Institute Technical University of Denmark Kgs Lyngby Denmark; ^3^ Plant Based Food and Beverages Department Novonesis Kongens Lyngby Denmark

**Keywords:** chickpea protein, in vitro bioaccessibility, protein functionality, protein–phenolic interaction, spent coffee phenolics

## Abstract

Plant‐based proteins exhibit low functionality compared to animal‐based counterparts. In this regard, protein–phenolic interaction is one of the promising methods for boosting the functional properties of proteins as well as improving the stability of phenolic compounds. This study evaluated the binding ability and the effect of interaction between phenolic extracts from spent coffee grounds, chosen to be a sustainable and abundant phenolic source, and chickpea protein isolate (CPI). Extracted phenolics were identified as chlorogenic acid, cryptochlorogenic acid, caffeic acid, and catechin. Also, the effect of interaction on the protein functionality and in vitro digestion properties of the protein‐phenolic complex was assessed. Different interaction conditions were tested, including varying concentrations of phenolic extract (PE) (0, 0.5, 1.0, and 1.5 mg/mL) at two different pH levels (pH 7.0 and 9.0, for better protein solubility) since these conditions affect the bond formation. The formation of the bond was also assessed to be affected by pH and concentration. Although there were no significant improvements in the protein solubility, the foaming and emulsifying properties of CPI were improved after its interaction with phenolic compounds. The highest improvements were observed for the emulsion activity index, emulsion stability index, and foaming capacity up to 71%, 82%, and 69%, respectively. On the other hand, the antioxidant properties of the protein‐phenolic complex before and after digestion were increased by 71% and 37%, respectively. The best interaction condition for antioxidant properties and protein functionality was found at pH 9.0 and the phenolic concentration of 1.5 mg/mL PE sample. This study demonstrated that the functionality of CPI can be improved by spent coffee phenolics; however, the conditions can be enhanced by the investigation of individual phenolic compounds and optimization of interaction conditions. With the improvement of protein functionality, CPI can be used in combination with phenolics from sustainable sources like spent coffee grounds in various plant‐based alternative foods as well as new product formulations.

## Graphical abstract

The bond formation was observed after the interaction between chickpea protein isolate (CPI) and spent coffee phenolics. Interaction improved the foaming and emulsion properties of CPI depending on the pH and concentration. Increase in phenolic concentration enhanced the antioxidant properties.

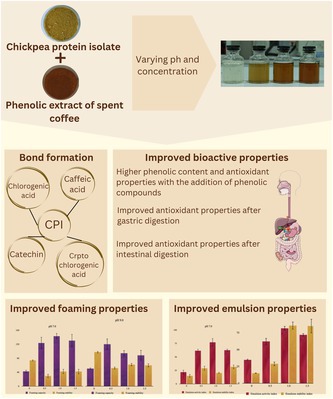

## 1 Introduction

Proteins are among the most vital elements of a human diet and are utilized extensively in the food industry for a variety of reasons, most notably their functional properties in food systems. Although many different protein sources having been utilized today, animal‐based proteins are still the most widely used ones due to their functional and nutritional properties. Nevertheless, they have a number of drawbacks, including the time‐consuming production process of animal‐based proteins and negative environmental effects mostly associated with carbon emissions (Wang et al. 2020). The use of animal‐based proteins might also be limited due to ethical concerns about animal welfare and religious beliefs. Considering their potential application in the food industry, plant‐based proteins are important alternative sources in this respect (Nikbakht Nasrabadi et al. 2021). In particular, chickpea, one of the most widely consumed legumes worldwide, has high protein content that is rich in essential amino acids together with lower off‐flavor contributors. In addition to these strengths, chickpea protein exhibits promising functional properties in particular foaming and emulsifying (Gundogan et al. 2024). However, these functional and nutritional properties of chickpea protein are not competitive with conventional animal‐based proteins, as is the case for most of the plant proteins such as pea, bean, and soy protein isolate/concentrates (Day et al. 2022). Numerous studies have been conducted so far to investigate and characterize the functional properties of chickpea protein isolates (CPI) as well as to explore the various possible modification approaches to enhance them (Gundogan et al. 2024). CPI can be structurally modified through diverse chemical, physical, or enzymatical treatments which might lead to improvement in functional properties due to protein structure alteration (Ramani et al. 2021; Zhang et al. 2023).

Phenolic compounds are secondary metabolites that possess many bioactive properties including antioxidant, antidiabetic, antihypertensive, and anti‐inflammatory activities (Ozdal et al. 2013). They can interact and form a bond with different components in complex food matrices, particularly with proteins. The reciprocal interaction between phenolic compounds and proteins is regarded as a protein modification method since this covalent interaction causes changes in the structural and functional properties of proteins (Nikbakht Nasrabadi et al. 2021); in addition, protein–phenolic interaction may improve the bioactivity and stability of phenolic compounds (Günal‐Köroğlu et al. 2023). Phenolic components might be introduced to the food matrices in purified forms or through inherently phenolic‐containing organic systems, such as fruits, that might be used as additional raw material/ingredients for co‐processing to further induce protein–phenolic interaction. Recently, there is a significant upsurge in the number of studies framing the potential of phenolic‐rich food materials such as berries as well as diverse by‐products (fruit peels, tea waste, oil seed by‐products, etc.) (Vidović et al. 2022; Çakmak et al. 2024; Ceylan et al. 2023). Particularly, phenolic‐rich food‐grade by‐products are of great importance due to their economic value and sustainability concerns (Ozkan et al. 2023).

Every year, around 6 million tons of spent coffee grounds, the insoluble part of the ground coffee after brewing, are generated since coffee is one of the most prefferred warm drinks worldwide (Monente et al. 2015; Janissen and Huynh 2018). A gram of spent coffee contains 10–17 mg of phenolic compounds (gallic acid equivalent; Choi and Koh 2017; Campos‐Vega et al. 2015). Hence, spent coffee grounds have a significant potential not just to possess generous amounts of residual phenolic compounds but also its superabundant daily production, globally. On top of these factors, ease of accessibility and no/low price make spent coffee grounds an excellent candidate that is worth investigating. Although there are some studies exploring the interaction between the coffee phenolics and proteins, studies regarding the interaction of spent coffee phenolics with proteins are very limited. Although some studies focus on the interaction between naturally occurring phenolics and proteins in coffee (Ali et al. 2012), the detailed interaction between spent coffee phenolics and chickpea protein in terms of structural alterations and binding kinetics has also been investigated for the first time by our research group recently (Saricaoglu et al. 2024; Shi et al. 2022). In addition to this, the interaction between plant‐based proteins and chlorogenic acid, one of the major phenolic compounds in spent coffee phenolics, has been reported as an important method to alter the structure of proteins and improve their functional properties (Shi et al. 2022; Tarahi et al. 2024).

In order to carry the existing knowledge a step further, this study aims to investigate the effect of the interaction between CPI and spent coffee phenolics on protein functionality and in vitro digestibility of the induced protein‐phenolic complexes. Interactions were carried out at different pH and phenolic concentrations. In this way, the effect of different interaction conditions of spent coffee phenolics and CPI was evaluated regarding protein‐phenolic binding, protein functionality, and in vitro digestion properties of the protein‐phenolic complex.

## 2 Materials and Methods

### 2.1 Materials

Chickpeas were kindly donated by Dervisoglu A.S (Mersin, Türkiye) and spent coffee grounds were gathered on three different days at a Starbucks coffee shop in Istanbul, Türkiye. All chemicals used during the study were of analytical purity and purchased from Sigma‐Aldrich unless specified otherwise. All the analyses were conducted using Milli‐Q water.

### 2.2 Proximate Composition Analyses

The proximate composition analyses were carried out on the chickpea flour and spent coffee grounds. The moisture content was determined by Infrared (Precisa, XM 50, Switzerland). AOAC Official Methods (AOAC 2003) were used to analyze ash (923.03), lipid (920.85) and crude protein (920.87) contents. The nitrogen conversion factor of 6.25 was used to calculate the protein content of chickpea flour and spent coffee grounds.

### 2.3 Isolation of Chickpea Protein

Protein samples were isolated using the method described by Karaca et al. (2011), with slight modifications. Samples were first ground using an analytical grinder (IKA A11 basic analytical mill). The powdered samples were then combined with hexane (1:1, w:v) and agitated for 30 min to remove fat from the sample. The mixture was filtered using filter paper (Whatman Gr. 1). After four repetitions of the procedure, samples were incubated overnight for residual hexane evaporation.

Defatted chickpea flour was combined with distilled water (1:10), and the pH was adjusted to 9.0 with 1.0 M NaOH. After 60 min of stirring at ambient temperature, the mixture was centrifuged at 8000 rpm for 15 min at 4°C using a Universal 320R centrifuge (Hettich, Westphalia, Germany). For protein precipitation, the pH of the supernatant was adjusted to 4.5 using 1.0 M HCl. After centrifugation, the protein was collected and kept at −20°C until it was freeze‐dried at −76°C for 24 h.

### 2.4 Phenolic Extraction From Spent Coffee Grounds

Spent coffee grounds (10 g) were combined with 120 mL of 75% ethanol. The mixture was placed in an ultrasonic bath (45 kHz, 120 W) for 15 min at room temperature and then centrifuged for 7 min at 9000 rpm and 4°C. To repeat the extraction, 80 mL of 75% ethanol was added to the residue after the supernatant was removed. After ultrasonic extraction and centrifugation, the supernatant was collected, and the extracts were pooled. Ethanol was removed using a vacuum (50°C) rotary evaporator system (IKA RV 10). The residual ethanol was eliminated by placing the extract under nitrogen for approximately 20 min. The residue was then freeze‐dried at −76°C for 36 h and stored at −18°C until further analysis.

### 2.5 Preparation of Protein‐Phenolic Complex

Protein isolate solutions (10 mg/mL) were prepared in distilled water at pH 7.0 and 9.0, then stored overnight at 4°C. The pH was adjusted using either 1.0 M HCl or NaOH solution. Phenolic extract solutions were prepared at three different concentrations as 0.5, 1.0, and 1.5 mg/mL. Immediately before analysis, protein and phenolic extract (PE) solutions were mixed at a ratio of 1:1 (v/v) and the pH of the solutions was readjusted to 7.0 or 9.0. Protein‐phenolic solutions were vortexed and then placed in an ultrasonic bath for 5 min. The control solutions were prepared with distilled water by adjusting pH at 7.0 and 9.0. Samples were prepared as fresh at the beginning of each analysis.

### 2.6 Determination of Binding Properties of Phenolic Compounds

The HPLC method was used to determine the phenolic compounds in the PE and protein‐phenolic complex according to the method of Capanoglu et al. (2008), with a W600 Waters HPLC system containing a photodiode array (PDA) detector. Since the HPLC system can only identify the free forms of phenolic compounds, the results of the PE and protein‐phenolic complex were compared in order to investigate the binding characteristics of phenolic compounds from spent coffee grounds.

### 2.7 Determination of Protein Functionality

#### 2.7.1 Protein Solubility

The protein solubility of the protein‐phenolic complex was determined by measuring protein content in the supernatant and protein solutions using the Bradford method (Bradford 1976). Protein‐phenolic solutions were centrifuged at 9000 rpm for 10 min before analysis. The results are expressed as mg bovine serum albumin per g protein‐phenolic complex. The protein solubility of samples was calculated by dividing the protein content of the supernatant by the protein content in the sample.

#### 2.7.2 Foaming Properties

The method of Wang et al. (2022), was followed with minor adjustments to determine the foaming capacity (FC) and foaming stability (FS) of protein‐phenolic complexes. A 15 mL of protein‐phenolic solution was homogenized at 8000 rpm for 2 min with a homogenizer (Ultra‐Turrax T18, IKA) to create foam structures. The foam volumes immediately after the homogenization and after 30 min of homogenization were used to calculate the FC and FS (Equations 1 and 2), respectively, as described by the following equations:
(1)

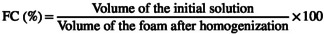



(2)

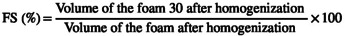




#### 2.7.3 Emulsion Capacity and Stability

The emulsion capacity of chickpea protein was evaluated by mixing 10 g of oil with 10 g of protein‐phenolic complex solution based on our previous trial to optimize the methodology (Appendix, Table S1). After the homogenization (Ultra Turrax T18) of the solution at 10,000 rpm for 5 min, the emulsions were divided into two tubes. The first solution was centrifuged at 6000 rpm for 2 min to measure emulsion capacity, and the second solution was centrifuged under the same conditions after being heated to 85°C for 15 min to measure emulsion stability. Six different sections of the emulsion were measured for height using a ruler. The average height of the emulsion was then divided by the total height of the solution and multiplied by 100.

#### 2.7.4 The Emulsion Activity Index and Emulsion Stability Index

The method of Pearce and Kinsella (1978) was followed to determine the emulsion activity index (EAI) and emulsion stability index (ESI). The same conditions described in Section 2.9 were used to prepare the emulsion. From the bottom of the emulsion, 50 μL of the sample was taken and added to 750 μL of phosphate buffer (pH 7.0, 10 mM) that included 0.1% SDS. A VWR (Avantor, USA) spectrophotometer was used to monitor the absorbance of solutions at 500 nm. After 10 min, the absorbance of the solution was recorded once again to calculate the emulsion stability index. Emulsion activity (Equation 3) and stability (Equation 4) indices were calculated using the equations below:

(3) $$\mathrm{EAI}\;\left(m^{2}/g\right)=\frac{2\cdot 2.303\cdot A_{0}\cdot N}{c\cdot \varphi \cdot 10,000}$$

(4) $$\mathrm{ESI}\;\left(\min\right)=\frac{A_{0}}{∆A}\cdot t$$

*A*_0_ is the absorbance value of diluted emulsion, *N* is the dilution factor, *c* is the weight of protein (g) per volume (mL), φ is the oil volume fraction, Δ*A* is the change in the absorbance after 10 min, and *t* is the incubation time (10 min).

### 2.8 In Vitro Gastrointestinal Digestion of Protein‐Phenolic Complex

In vitro gastrointestinal digestion of protein‐phenolic complex was determined by simulating the gastrointestinal digestion system; therefore, oral, gastric, and intestinal solutions were prepared according to the method by Minekus et al. (2014). Digestion was performed for oral, gastric, and intestinal phases at 37°C during 2 min for the oral phase and 2 h for the gastric and intestinal phases, individually. After the simulation of the gastrointestinal system, initial and digested samples were analyzed for total phenolic content and total antioxidant capacity.

### 2.9 Determination of Total Phenolic Content

Total phenolic content was determined using the method by Singleton and Rossi (1965) and results were expressed as mg gallic acid equivalent per g sample.

### 2.10 Determination of Total Antioxidant Capacities

Two methods, including ABTS (2,2′ azinobis (3‐ethylbenzothiazoline‐6‐sulfonic acid) diammonium salt) and CUPRAC (Cupric reducing antioxidant capacity) were performed to analyze the total antioxidant capacities of initial (undigested) and digested samples. The ABTS method was performed according to the procedure by Miller and Rice‐Evans (2009), and the CUPRAC method was performed according to the procedure by Apak et al. (2009). Results were expressed as mg Trolox equivalent per g sample.

### 2.11 Statistical Analysis

Separate extractions were performed on the spent coffee grounds collected on three different days and analyzed as triplicate. The results were expressed as a mean ± standard deviation of these measurements. SPSS software was used for the statistical analysis (IBM SPSS Statistics for Macintosh, Version 27.0). One‐way ANOVA was used to analyze statistical differences between means at a significance level of 5%.

## 3 Results and Discussion

### 3.1 Material Characterization

Proximate analysis was carried out on the raw material including chickpea flour and spent coffee grounds (Table 1). On a wet basis (10.03% ± 0.11% moisture), the protein content of defatted chickpea flour was 23.36% ± 0.42% while the protein content of CPI was determined as 89.97% ± 0.78%. Despite the fact that proximate content might vary depending on a number of variables, including crop variety, environmental and agricultural conditions, research published in the literature has consistently provided similar findings. For example, Kaur and Singh (2007) discovered that the protein isolates obtained from various chickpea flours (defatted, 20%–26% protein) by the isoelectric precipitation method had a protein content range between 89% and 94%. On the other hand, some studies showed that the chickpea protein might yield a lower protein content despite the application of the same isolation method which might be related to the initial protein and fat content of chickpea.

**TABLE 1:** Proximate composition of de‐fatted chickpea (DCF) flour and spent coffee grounds (SCG).

Composition	DCF % (w/w)	SCG % (w/w)
Moisture	10.03 ± 0.11	59.59 ± 1.86
Protein	23.36 ± 0.42	6.92 ± 0.39
Ash	2.56 ± 0.01	0.97 ± 0.01
Fat	5.74 ± 1.29	6.91 ± 0.43
Total Carbohydrate	55.46 ± 0.43	25.61 ± 1.76

*Note:* The results are represented as mean ± standard deviation (*n* = 3).

The proximate composition of spent coffee grounds is provided in Table 1, indicating a specific distribution on a wet basis (59.59% ± 1.86% moisture). The proximate composition of spent coffee grounds can be expected to vary considerably, as it can be obtained from many different producers and roasteries with diversified blends or after different kinds of coffee preparation/brewing processes. For instance, spent coffee ground was declared to consist of 2.10%–2.95% moisture, 8.58%–18.55% oil, 13.87%–16.12% protein, and 61.76%–70.37% carbohydrates on a dry basis (Bijla et al. 2022). Due to extreme moisture level differences, it is challenging to make a concrete comparison and discussion with the current literature data and our results; however, it is already clear that they are quite different, for instance in terms of their fat content: our sample has a fat content around 7% together with almost 60% moisture, whereas literature data shows a similar value (8.5%) despite having almost 3% moisture content.

### 3.2 Binding Properties of Phenolic Compounds

Mainly four phenolic compounds were identified in PE and protein‐phenolic complexes including chlorogenic acid, cryptochlorogenic acid, caffeic acid, and catechin (Table 2). Due to the fact that only the free state of phenolic compounds was detected, the inability to detect some of the phenolic compounds was associated with a potential protein binding. Consequently, although PE contains chlorogenic acid, neither the samples interacted at pH 7.0 nor the interaction CPI + PE 0.5 sample at pH 9.0 revealed the presence of chlorogenic acid. This was associated with the bond formed between chlorogenic acid and chickpea protein. Similarly, a bond formed between the cryptochlorogenic acid and chickpea protein at both interaction conditions. The concentration of cryptochlorogenic acid was decreased with the increase in phenolic concentration at the interaction that occurred at pH 7.0. Similar to our findings, studies also showed that the increase in the phenolic concentration may increase the number of bounded phenolic compounds in the protein (Jiang et al. 2019). The chlorogenic acid, caffeic acid, and catechin concentrations in the CPI + PE 0.5 sample, which interacted at pH 9.0, were dramatically decreased. The decrease in the concentration of phenolic compounds indicates their binding to proteins also depends on the type and structure of phenolic compounds, as similarly reported in the literature (Yildirim‐Elikoglu and Erdem 2018; Tian et al. 2023). Although non‐covalent interactions are more likely to occur at pH 7.0, covalent interactions are more probable at pH 9.0 (Yildirim‐Elikoglu and Erdem 2018; Zhang et al. 2021). A complex formed with a covalent bond is more stable than another one formed with a non‐covalent bond since it is more difficult to break the covalent bond. The lower concentration of phenolic compounds in the interaction conditions at pH 9.0 can be related to the fact that the phenolics are able to create a more stable bond at pH 9.0 than at pH 7.0 (Liu et al. 2019). In our previous study, the in‐depth interaction between chickpea protein and spent coffee phenolics was examined, focusing on the binding properties through fluorescence spectroscopy, recently (Saricaoglu et al. 2024). As observed in this study, more stable bonds were formed between CPI and cryptochlorogenic acid, caffeic acid, and catechin at pH 9.0, which indicates that a covalent bond can be formed between them.

**TABLE 2:** Binding properties of protein‐phenolic complexes.

pH	Material	Chlorogenic acid	Cryptochlorogenic acid	Caffeic acid	Catechin
—	PE	4.23 ± 0.46	5.57 ± 0.6^a,⟨^	5.93 ± 0.34^a,⟨^	20.95 ± 1.84^a,⟨^
7.0	CPI + PE 0.5	—	5.53 ± 0.47^a^	4.49 ± 0.56^b^	20.65 ± 2.11^a^
	CPI + PE 1	—	5.09 ± 0.87^ab^	4.19 ± 0.74^b^	18.97 ± 5.29^a^
	CPI + PE 1.5	—	4.23 ± 0.22^b^	4.73 ± 0.30^b^	21.65 ± 1.82^a^
9.0	CPI + PE 0.5	—	2.82 ± 0.30^†^	1.37 ± 0.16^†^	15.09 ± 1.45^†^
	CPI + PE 1	5.26 ± 0.28	5.06 ± 0.40^⟨^	4.41 ± 0.56^|^	18.84 ± 3.80^⟨^
	CPI + PE 1.5	5.40 ± 0.19	5.62 ± 0.44^⟨^	4.65 ± 0.43^|^	19.66 ± 4.20 ^⟨^

*Note:* The results are represented as mean ± standard deviation (*n* = 3). CPI: Chickpea protein isolate; PE: Phenolic extract. The results are given in mg phenolic compound/g sample. Different letters (a, b, and c) indicate significant differences between different concentrations of the interaction occurred at pH 7.0 (*p* < 0.05). Different letters (⟨, †, and |) indicate significant differences between different concentrations of the interaction that occurred at pH 9.0 (*p* < 0.05).

### 3.3 Protein Solubility

**FIGURE 1:**
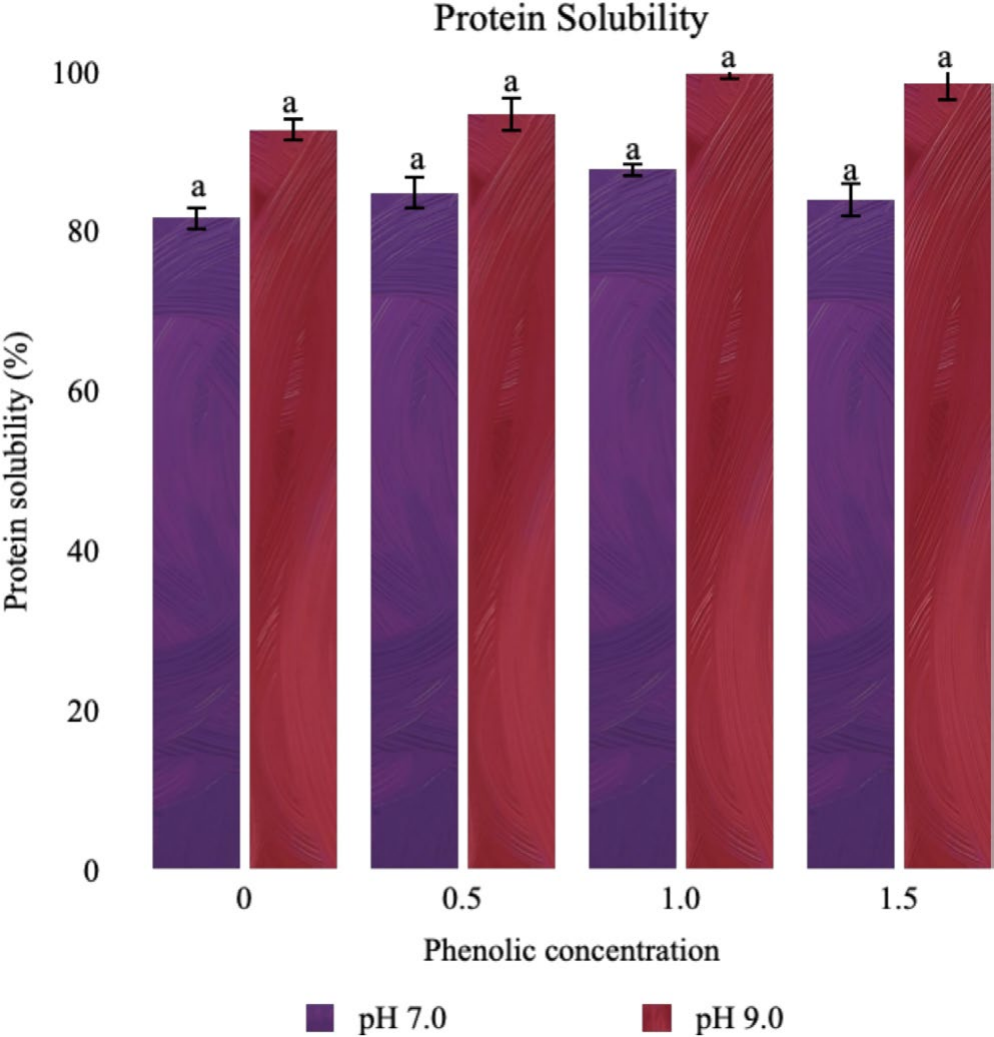
Protein solubility of protein‐phenolic complex.

Due to the fact that insoluble proteins cannot exhibit any functional properties, protein solubility is one of the most important variables in displaying their functional properties. Change in the protein solubility of CPI and the protein‐phenolic complex is shown in Figure 1. The solubility of CPI was determined as 81.72% at pH 7.0 and 92.76% at pH 9.0, whereas similar values were reported by Karaca et al. (2011), who discovered the CPI solubility to be 91.20% at pH 7.0. Although there was an incremental tendency in protein solubility after the interactions with phenolics at pH 7.0 and pH 9.0, it was detected to be statistically insignificant (*p* > 0.05). This finding points out that the interaction of CPI with spent coffee phenolics did not affect the solubility of the protein, as expected.

Phenolic compounds may affect the solubility of proteins by attaching to proteins' surfaces and modifying their surface properties, changing their surface hydrophobicity (Liu et al. 2019). As a result of their interaction with phenolics, a change in the solubility characteristics of the protein is mostly predicted, either positively or negatively. The predominant phenolic ingredient in spent coffee, chlorogenic acid, has been demonstrated in studies to improve the solubility of casein and whey protein isolate (Jiang et al. 2018) while decreasing the solubility of bovine serum albumin, lysozyme, and α‐lactalbumin (Prigent et al. 2007, 2003). Even while these fluctuations in solubility are associated with a change in protein structure, in some cases, the solubility may not change despite the alteration in protein structure (Prigent et al. 2003). The observed data in our study seem consistent with this phenomenon in the literature; the solubility did not change after the phenolic interaction, which can be associated with the soluble phenolic compounds that may affect the protein solubility (Fernando and Manthey 2022). In addition to this, the increase in the protein solubility is expected with the increase of pH. However, the nonsignificant solubility difference might be further explained by the repositioning of protein hydrophobic sites on the surface at pH 9.0 more than that of pH 7.0 (Saricaoglu et al. 2024).

### 3.4 Foaming Properties

**FIGURE 2:**
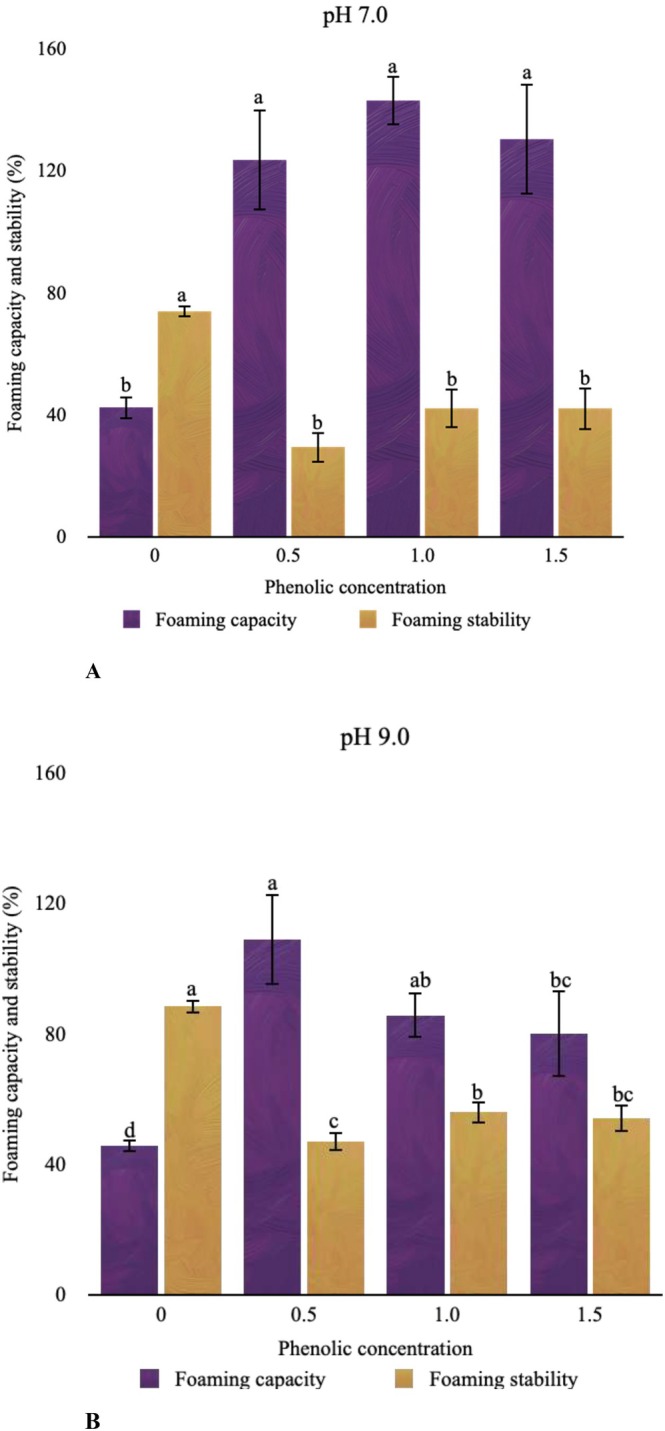
Foaming capacity and foaming stability of protein‐phenolic complex interacted at pH 7.0 (A) and pH 9.0 (B).

FC and FS are related to the ability of protein to form a layer for trapping and keeping the air inside, and this property is affected by some critical factors like the solubility, hydrophobicity, and flexibility of proteins (Zayas 1997). The foaming properties of CPI and its phenolic complexes are presented in Figure 2. The foaming capacities of CPI were 43.33% and 45.56% at pH 7.0 and 9.0, respectively, with no significant differences observed between pH values (*p* > 0.05). On the other hand, the effect of protein concentration on the foaming properties was insignificant under the pH 7.0 condition, whereas different results were obtained with the concentration change at pH 9.0. The phenolic interaction improved the FC of CPI; on the contrary, the FS decreased after its interaction with PE at both pH conditions. The best condition regarding foaming properties can be proposed as the CPI + PE 0.5 sample at pH 9.0. Improved FC can be associated with increased protein solubility following phenolic interaction, which results in an increase in the amount of protein that contributes to functionality and enhances protein unfolding (Li et al. 2020). Unfortunately, our findings can not be explained by this pervasive behavior of solubility‐functionality relation. However, the observed foamability increase might be attributed to alterations in the CPI, as hydrophobic peptides, which are mainly found in the protein's interior regions, relocate towards the surface during protein–phenolic interaction, resulting in an increase in FC. Similarly, a decrease in the fluorescence intensity of CPI after its interactions with spent coffee phenolics was reported in our previous study, demonstrating a correlated increase in the protein hydrophobicity and improved foaming properties (Saricaoglu et al. 2024). However, the poor stability indicates that the structure cannot be maintained, which might be connected with an increase in interfacial tension as a result of protein–phenolic interaction (Parolia et al. 2022). In addition, several non‐covalent interactions, including hydrogen bonding and van der Waals forces, particularly observed around pH 7.0, cannot retain their stability in a non‐buffered aqueous environment.

### 3.5 Emulsifying Properties

**FIGURE 3:**
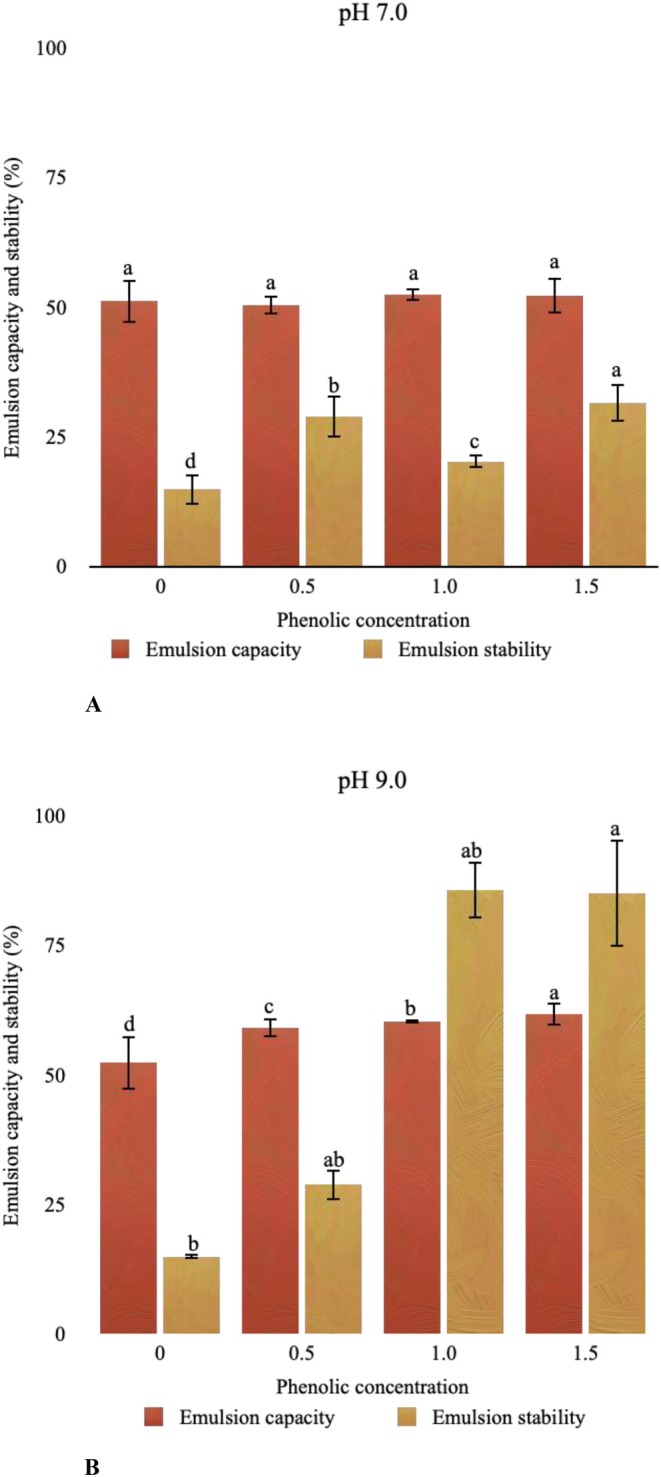
Emulsion capacity and stability and emulsion activity and emulsion stability index of protein‐phenolic complex interacted at pH 7.0 (A) and pH 9.0 (B).

When the influence of phenolic interaction on emulsion capacity and stability was investigated, different results were obtained from the samples at pH 7.0 and pH 9.0. Interaction performed at pH 7.0 had no significant effect (*p* > 0.05) on the emulsion capacity of CPI, whereas little difference in emulsion stability was observed (Figure 3). On the other hand, an increase in emulsion capacity and stability was detected with the increasing phenolic concentration performed at pH 9.0 (*p* < 0.05). In particular, both emulsion capacity and stability increased by almost 10% with the addition of PE at the sample of CPI + PE 1.5 under pH 9.0 conditions.

**FIGURE 4:**
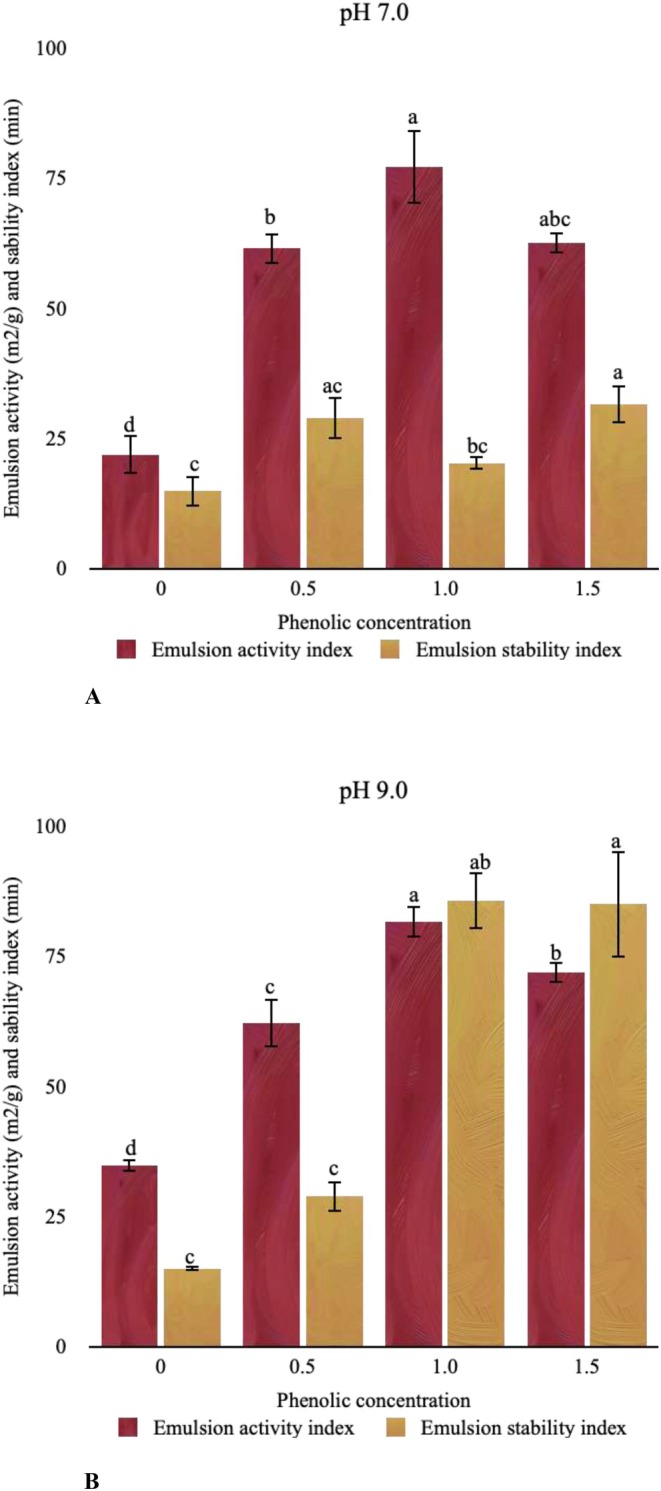
Emulsion activity and emulsion stability index of protein‐phenolic complex interacted at pH 7.0 (A) and pH 9.0 (B).

Protein–phenolic interaction exhibited a positive effect on the EAI and ESI of CPI at both pH environments (Figure 4). The EAI and ESI increased depending on the phenolic concentration. The highest EAI (77.10 ± 7.03 m^2^/g) was measured for the sample of CPI + PE 1.0 (71%) whereas the highest ESI (31.59 ± 3.64 m^2^/g) was measured for the sample of CPI + PE 1.5 (53%) following the interaction performed at pH 7.0. Similar results were obtained for the pH 9.0 interactions. Considering the interaction conditions, the concentration of the phenolics pioneered as the main driving force. Although there was no significant difference detected between the two interaction conditions, the EAI and ESI of the samples CPI, CPI + 0.5, CPI + 1.0, and CPI + 1.5 were significantly different from each other.

The nonpolar groups of the proteins are responsible for water holding, whereas polar groups are responsible for oil holding (Zayas 1997). The surface properties of the protein, or the ratio of nonpolar to polar amino acids, should be proportional to the emulsion's composition. The improvement of surface properties is the most important assumption for the interaction of phenolic compounds with proteins in terms of emulsifying properties (Zayas 1997). This improvement is mostly associated with the altered structure of the protein after phenolic interaction. The surface properties and hydrophobic environment‐related capabilities may differ after phenolic interaction. Although phenolic addition to emulsions has been shown to have adverse effects on emulsifying properties in certain studies (Günal‐Köroğlu et al. 2022; Malik and Saini 2017), some studies found that phenolic addition might have positive effects on the emulsion properties of proteins, which is in good association with this present work. Specific phenolic compounds that have been shown to have positive effects on the emulsifying properties of plant proteins include chlorogenic acid (Pan et al. 2019; Karefyllakis et al. 2017) and PEs, such as those found in flaxseed (Pham et al. 2019).

### 3.6 In Vitro Gastrointestinal Digestion

So far, many studies reported that protein–phenolic interaction affects the digestive properties and antioxidant capacities of phenolic compounds (Yildirim‐Elikoglu and Erdem 2018). The effect of protein–phenolic interaction on the total phenolic content and total antioxidant capacity values of CPI‐PE complexes at the different stages of digestion is presented in Table 3. The total phenolic content and antioxidant capacities of protein‐phenolic complexes mostly increased with the increase of phenolic concentration at the initial stage, in contrast to the majority of studies that indicated a decrease in the total antioxidant capacities after protein–phenolic interaction (Yildirim‐Elikoglu and Erdem 2018). Different antioxidant capacity trends were observed after gastric digestion. The total antioxidant capacities of protein‐phenolic complexes increased with the increase in phenolic concentration measured by the CUPRAC method. On the other hand, protein‐phenolic complexes had lower total antioxidant capacities compared to the CPI alone after gastric digestion measured by the ABTS method. This can be associated with the variation of antioxidant potential of different bioactivities in different methods since they are based on varying chemical reactions (Christodoulou et al. 2022).

**TABLE 3:** In vitro digestion properties of protein‐phenolic complexes.

Phase	Sample	Interaction at pH 7.0			Interaction at pH 9.0		
		ABTS	CUPRAC	TPC	ABTS	CUPRAC	TPC
Initial	CPI	11.95 ± 0.47^d,A^	13.30 ± 2.95^d,A^	13.21 ± 0.48^d,A^	19.00 ± 2.82^b,B^	12.02 ± 1.35^d,A^	12.94 ± 0.22^d,A^
	CPI + PE 0.5	19.44 ± 0.75^c,A^	27.52 ± 4.77^c,A^	20.91 ± 1.03^c,A^	18.83 ± 0.66^b,A^	21.33 ± 4.20^c,B^	17.14 ± 0.49^c,B^
	CPI + PE 1.0	22.26 ± 1.57^b,A^	34.81 ± 5.29^b,A^	27.12 ± 1.76^b,A^	17.61 ± 0.27^b,B^	38.54 ± 5.81^b,A^	22.53 ± 0.65^b,B^
	CPI + PE 1.5	42.18 ± 2.80^a,A^	44.69 ± 6.09^a,A^	29.55 ± 1.12^a,A^	32.94 ± 0.93^a,B^	40.82 ± 2.21^a,B^	25.69 ± 1.16^a,B^
Gastric	CPI	87.85 ± 1.53^a,A^	56.85 ± 6.73^c,A^	58.01 ± 8.06^a,A^	72.77 ± 1.85^a,B^	13.31 ± 4.22^c,B^	46.38 ± 4.63^a,B^
	CPI + PE 0.5	82.03 ± 0.88^b,A^	68.12 ± 2.26^bc,A^	62.00 ± 1.98^ac,A^	66.54 ± 3.48^b,B^	19.67 ± 4.23^c,B^	45.53 ± 1.90^a,B^
	CPI + PE 1.0	75.76 ± 0.99^c,A^	71.71 ± 6.32^b,A^	68.81 ± 2.27^bc,A^	64.60 ± 3.21^cb,B^	30.82 ± 6.56^b,B^	46.35 ± 3.83^a,B^
	CPI + PE 1.5	71.49 ± 0.67^d,A^	95.51 ± 6.91^a,A^	64.92 ± 2.50^ac,A^	58.35 ± 1.25^d,B^	37.14 ± 5.53^ab,B^	46.40 ± 1.62^a,B^
Intestinal	CPI	162.31 ± 6.48^a,A^	115.81 ± 8.24^c,A^	77.71 ± 3.90^a,A^	118.39 ± 3.97^d,B^	53.73 ± 8.67^ab,B^	56.63 ± 2.79^a,B^
	CPI + PE 0.5	152.75 ± 2.82^b,A^	145.53 ± 8.67^bc,A^	75.26 ± 3.50^a,A^	144.59 ± 3.83^c,B^	48.36 ± 4.72^b,B^	49.03 ± 2.59^b,B^
	CPI + PE 1.0	142.29 ± 1.23^c,A^	152.66 ± 7.91^ba,A^	73.31 ± 3.44^a,A^	177.33 ± 3.84^a,B^	54.99 ± 8.63^b,B^	45.67 ± 5.50^cb,B^
	CPI + PE 1.5	133.73 ± 1.22^d,A^	184.54 ± 9.00^a,A^	72.54 ± 2.91^a,A^	161.07 ± 3.66^b,B^	65.60 ± 6.12^a,B^	49.52 ± 2.20^db,B^

*Note:* The results are represented as mean ± standard deviation (*n* = 3). CPI: Chickpea protein isolate; PE: Phenolic extract. Different lowercase letters indicate significant differences between different concentrations of the same sample group (*p* < 0.05). Different uppercase letters indicate significant differences between different pH values of the sample in the same concentration. The results for ABTS and CUPRAC methods were given as mg Trolox equivalent per g sample. The results for the TPC method were given as mg gallic acid equivalent per g sample.

After the intestinal phase, the phenolic content of samples was decreased with the phenolic interaction. During gastrointestinal digestion, the protection of phenolic compounds from degradation and oxidation is expected due to their complex formation with proteins. In our study, significant increases were observed in the antioxidant capacities of all samples, including control, after intestinal digestion compared to gastric digestion. However, CPI alone (control) at pH 7.0 exhibited the highest antioxidant capacity measured by the ABTS method among all the samples. This can be related to the degradation of present phenolic compounds during gastrointestinal digestion (Chen et al. 2019). In addition to this, digestive enzymes may interact with phenolic compounds, and this may decrease the availability of phenolic compounds as well as the digestibility of the protein. As a result of this, the antioxidant capacity of the protein‐phenolic complex might decrease (Cirkovic Velickovic and Stanic‐Vucinic 2018). The protein‐phenolic complex may have a less stable bond structure at pH 7.0 compared to pH 9.0 and thus phenolic compounds become free before the intestinal digestion and lose their activity since they are not stable in the digestive system (Ozdal et al. 2013). Besides, proteins, in particular, peptides, may exhibit antioxidant properties. The highest level of antioxidant capacity measured by the ABTS method can be associated with an increase in the antioxidant properties of CPI after digestion. Similar results were reported by Zhao et al. (2020) which indicated that the antioxidant capacity of gallic acid and casein complex decreased after digestion due to the digestive enzymes in the gastrointestinal tract and poor stability of phenolic compounds during digestion.

So far, studies in the literature have discovered that when phenolic compounds are attached to proteins, free hydroxyl groups can generate antioxidant potential, boosting the overall antioxidant capacity of protein‐phenolic complexes. Casein and gallic acid complexes, as well as complexes of chlorogenic acid with whey protein and casein, have been reported to have increased total antioxidant capacities (Zhao et al. 2020). On the other hand, the stability of interaction, phenolic degradation, pH, catalysis of protein, and formation of different peptides may affect the antioxidant properties of protein‐phenolic complexes after in vitro digestion.

## 4 Conclusion

Protein–phenolic interaction affects the functional properties of proteins by changing their structural properties. On the other hand, due to the complex formation, proteins may improve the bioactive properties of phenolic compounds by increasing their stability during gastrointestinal digestion. In this study, the effect of interaction between spent coffee PE and CPI occurred at pH 7.0 and 9.0 on the protein functionality of phenolics, and in vitro digestion properties of the protein‐phenolic complex were demonstrated. Covalent bond formation is induced between CPI and phenolic compounds in PE from spent coffee grounds including chlorogenic acid, cryptochlorogenic acid, caffeic acid, and catechin. Besides, the binding of CPI to the phenolic compounds was affected by phenolic concentration and pH. As a result of this interaction, the functional properties of CPI and its phenolic complexes were altered. Significant improvements were observed for emulsion and foaming properties, indicating that spent coffee PE improved the interfacial properties of CPI. Besides, the antioxidant properties were mostly improved after in vitro digestion. In particular, the best condition observed was at pH 9.0 and the concentration of 1.5 mg/mL PE sample in terms of protein functionality and antioxidant properties. However, the interaction conditions are significantly affected by the in vitro digestion properties of the protein‐phenolic complex. Besides, varying antioxidative properties were an indicator of the change in the phenolic composition since phenolic compounds may react distinctively to different antioxidant activity detection methods. From a general viewpoint, CPI and spent coffee PE interaction is not an ideal way of modification to increase antioxidant properties alone, since the control sample has the highest performance. However, this interaction might improve the functional properties together with high antioxidant properties, whereas the control sample does not perform functionality as promisingly as phenolic interacted samples. Improved functionality comes with a price of relatively reduced antioxidant properties followed by in vitro digestion.

Consequently, spent coffee PE induced a promising improvement for the protein functionality of CPI. Although the matrix effect can be important, the interaction of individual phenolic compounds needs to be further investigated since it may improve the effect of interaction. In particular, the effect of cryptochlorogenic acid, caffeic acid, and catechin should be individually analyzed since they might form more stable bonds with chickpea protein.

## Author Contributions

**Beyza Saricaoglu:** conceptualization (supporting), formal analysis (lead), investigation (lead), writing – original draft (lead). **Busra Gultekin Subasi:** conceptualization (supporting), methodology (supporting), writing – review and editing (equal). **Esra Capanoglu:** conceptualization (lead), supervision (lead), writing – review and editing (lead).

## Supporting information


**Table S1:** Emulsion capacity and stability of chickpea protein isolate at different pH values and oil ratios.

## Data Availability

Data available on request from the authors.
