# Supplementary material for: Sustainable Augmentation of Chickpea Protein Functionality and In Vitro Digestion via Spent Coffee Phenolics: A Protein–Phenolic Interaction Study
Source: Food Sci Nutr. 2025 Nov 21;13(11):e70914. doi: 10.1002/fsn3.70914 (PMC12639185; doi:10.1002/fsn3.70914)
Supplement: Supplementary file 1 — Table S1: Emulsion capacity and stability of chickpea protein isolate at different pH values and oil ratios. [file FSN3-13-e70914-s001.docx]

| Oil ratio | pH 7.0 | | pH 9.0 | |
| --- | --- | --- | --- | --- |
|  | Capacity | Stability | Capacity | Stability |
| 10% | 12.95±1.38 | 10.41±1.51 | 10.11±1.41 | 13.94±3.48 |
| 20% | 18.98±0.78 | 16.78±1.32 | 16.53±0.55 | 16.60±8.98 |
| 30% | 27.47±0.55 | 26.82±0.37 | 25.26±1.47 | 29.72±2.25 |
| 40% | 39.06±5.52 | 32.55±1.47 | 35.94±0.74 | 34.10±2.18 |
| 50% | 54.52±1.60 | 44.15±2.91 | 51.23±4.36 | 45.34±0.30 |

**Table 1S.** Emulsion capacity and stability of chickpea protein isolate at different pH values and oil ratios
